# First report of a species of *Senga* (Cestoda: Bothriocephalidae) in commercial *Betta splendens* (Actinopterygii: Osphronemidae) in Brazil

**DOI:** 10.1007/s11259-026-11467-y

**Published:** 2026-08-18

**Authors:** Mariana Rodrigues Vale, Ana Clara Fernandes de Moraes, Sarah Portes Carneiro, Felipe Henrique Teixeira da Mata, Jordana Costa Alves de Assis, Henrique César Pereira Figueiredo, Matheus Anchieta Ramirez, Hudson Alves Pinto, Guilherme Campos Tavares

**Affiliations:** 1 Department of Preventive Veterinary Medicine, School of Veterinary Medicine, Federal University of Minas Gerais (UFMG), Belo Horizonte, Minas Gerais Brazil; 2 Department of Animal Science, School of Veterinary Medicine, Federal University of Minas Gerais (UFMG), Belo Horizonte, Minas Gerais Brazil; 3 Department of Parasitology, Institute of Biological Sciences, Federal University of Minas Gerais (UFMG), Belo Horizonte, Minas Gerais Brazil

**Keywords:** Ornamental fish, Helminths, Cestodes, Disease

## Abstract

Species of the genus *Senga* comprise a group of bothriocephalidean cestodes that predominantly parasitize freshwater teleost fishes, causing severe histopathological alterations due to scolex attachment to the digestive tract or obstruction of the intestinal lumen. The Siamese fighting fish *Betta splendens* is the type-host of *Senga besnardi*, the type species of the genus known only from the original description. Despite the importance of this fish species to ornamental aquaculture in Brazil, there are no prior records of this parasite in the Americas. Therefore, the present study aims to report the finding of *B. splendens* naturally infected with a bothriocephalidean cestode in a commercial fish farm from Brazil. In July 2024, four specimens of *B. splendens* exhibiting distension of the coelomic cavity were collected from an ornamental fish farm located in Patrocinio do Muriaé, Minas Gerais State. The animals were euthanized and submitted to parasitological analysis. Cestodes found in the fish were fixed, stained, and mounted for study by optical microscopy, with the aim of taxonomic identification. Cestodes were observed in the intestine of 3 out of 4 evaluated *B. splendens*, with infection intensities of 1, 8 and 10 cestodes per host. Morphological and morphometric analyses revealed that these specimens are indistinguishable from *S. besnardi*, particularly in the scolex morphology. However, given the complex taxonomy and the lack of molecular data for the species, the cestodes reported here are tentatively identified as *Senga* sp. This study represents the first report of *Senga* sp. infecting ornamental fish in the American continent.

## Background

Among the groups of fish pathogens important in aquaculture, cestodes represent a cosmopolitan group of flatworms that can parasitize teleosts both in the adult stage, within the host digestive tract, and in the larval stage, across various internal organs (Scholz and Kuchta 2017). This group of parasites is considered one of the most challenging pathogens in aquaculture, exerting severe economic impacts on the ornamental fish supply chain, where aesthetics dictates commercial value (Shinn et al. 2015). Considering species that use fish as definitive hosts, representatives of the order Bothriocephalidea deserve special mention. These cestodes possess complex life cycles that obligatorily rely on copepods as intermediate hosts, and the abundance of these hosts in fertilized tanks enhances larval transmission (Marcogliese 1995; Scholz 1997). The resulting pathology includes distension of the coelomic cavity, intestinal obstruction, hepatic necrosis, and severe nutritional loss, thereby affecting fish growth (Kuchta et al. 2018; Scholz and Kuchta 2025). The dispersion of such pathogens through the transport of infected batches compromises global biosecurity (Scholz and Kuchta 2025), affects production hubs, and results in significant production losses, necessitating rigorous sanitary control protocols.

The genus *Senga* Dollfus, 1934 comprises a group of bothriocephalidean that predominantly parasitize freshwater teleosts. In parasitized animals, infection with species of *Senga* causes severe histopathological alterations in the digestive tract. These include compression and atrophy of the intestinal mucosa, chronic inflammation with inflammatory cell infiltration at the scolex attachment site, and, in cases of high parasite loads, mechanical obstruction of the intestinal lumen. Such conditions can lead to emaciation, reduced growth rates, and increased susceptibility to secondary infections (Hassan et al. 2019; Padvi et al. 2024). Their main hosts include species from the families Channidae, Mastacembelidae, and Cyprinidae (Kuchta et al. 2008). However, their occurrence in the Siamese fighting fish *Betta splendens* (Regan, 1910) (Osphronemidae) represents a historical milestone in their dissemination via the ornamental trade (Dollfus 1934; Deshmukh 2016). Historically, the first species of this genus, *Senga besnardi*, was originally identified by Dollfus (1934) based on four worms found in two specimens of *B. splendens* maintained in a tropical aquarium from Paris, France. Since then, records of *Senga* spp. have been concentrated primarily in Asian and African countries, including Sierra Leone, India, China, Thailand, Pakistan, Singapore, and Malaysia, as well as in Australia (Kuchta et al. 2008; Bhure et al. 2010; Dhole et al. 2011; Madan et al. 2026). In the Americas, a previous report identified as cestodes from fish in Brazil as *Senga* sp., but a more recent study revealed that the agent corresponded to a new genus and species (Rego 1997; Scholz et al. 2017). Another study also identified as *Senga* sp. cestodes found in native fish in the same country (Azevedo et al. 2007), but no morphological support was provided and a definitive confirmation is still necessary.

*Betta splendens*, a species native to Southeast Asia (Indochina), stands out as one of the most appreciated and economically significant species in global ornamental aquaculture, serving as a pillar of the trade in aquatic organisms due to its phenotypic diversity (Zhang et al. 2022). The main parasitic diseases reported in *B. splendens* include ichthyophthiriasis and oodiniosis, as well as infestations by ciliated protozoans and monogeneans (Thilakaratne et al. 2003; Manklinniam et al. 2025). These agents, combined with secondary bacterial infections and the presence of invasive helminths such as bothriocephalidean cestodes, severely compromise the epithelial integrity and immunological status of the specimens (Noga 2010). Despite the taxonomic relevance of this record and the widespread dispersion of bothriocephalideans through international trade (Casanova-Hernández et al. 2025), there are still no formal scientific descriptions confirming the presence of species of the genus *Senga* in farmed fish within Brazilian territory or even in the American continent.

In the present study, adult cestodes obtained from *B. splendens* reared in a commercial farm in Brazil were subjected to morphological characterization for taxonomic identification.

## Case presentation

In July 2024, a visit was conducted to a fish farm located in the municipality of Patrocínio do Muriaé (21°09′10″S, 42°12′54″W), Minas Gerais state, Brazil. The property relies exclusively on family labor and engages in cattle ranching, although ornamental fish aquaculture is its primary economic activity. The fish are reared in greenhouses using masonry tanks lined with plastic sheeting. The water supply is sourced from an artesian well. During water exchanges, agricultural limestone and organic fertilizer (poultry litter) are applied to the tanks.

Four male specimens of *B. splendens* (1.825 ± 0.128 g, approximately 7 months old) exhibiting coelomic cavity distension were collected using a nylon hand net. The fish were transported alive in an oxygenated plastic bag to the Laboratory of Aquatic Animal Diseases (AQUAVET) at the Veterinary School of the Federal University of Minas Gerais (UFMG), Belo Horizonte, Minas Gerais state, Brazil, where they were euthanized via spinal transection using a scalpel, in accordance with CFMV Resolution No. 1000/2012 (Conselho Federal de Medicina Veterinária 2012) and subjected to parasitological examination.

The parasitological analysis involved the microscopic examination of fresh wet mounts prepared from epithelial mucus, gill tissue, and the digestive tract (Leibowitz et al. 2019). The samples were observed under a light microscope (Primo Star, Zeiss, Germany). Any recovered parasites were isolated, quantified, and fixed in 70% ethanol within Falcon tubes. For the morphological characterization, specimens of the parasites were stained with acetic alum carmine, dehydrated through a graded ethanol series, and cleared in beechwood creosote. The specimens were then permanently mounted on slides using Canada balsam (Pinto and Melo 2011). For analyses of the hooks of the apical disc, a scolex was cleared in Amman’s lactophenol and mounted on a non-permanent slide. These slides were examined under a light microscope (Leica DM750, Leica Microsystems, Germany) equipped with a digital camera ICC 500 (Leica Microsystems). The captured images were analyzed using LAS EZ software, version 3.4.0 (Leica Microsystems) and measures were obtained. Morphological and morphometric characteristics were compared with established taxonomic keys and descriptions by different authors (Dollfus 1934; Fernando and Furtado 1963; Khalil et al. 1994; Rego 1997). Voucher specimens were deposited at the Helminthological Collection of the Oswaldo Cruz Institute, Rio de Janeiro, Brazil (CHIOC 41109a-e). Attempts to obtain genetic sequences for the genetic markers 28 S rDNA (primers LSU and 1500R of Olson et al. 2003) and *cox*1 (primers JB3 and JB4.5 of Bowles et al. 1992), both widely used in the molecular characterization of parasitic platyhelminths, were unsuccessful.

Parasitological examination revealed the presence of cestodes in the intestine of three out of the four sampled specimens of *B. splendens*, resulting in a prevalence of 75%. A total of 19 worms measuring about 1.5 cm were recovered, and the intensity of infection found in each infected fish was 1, 8 and 10 specimens.

**Fig. 1:**
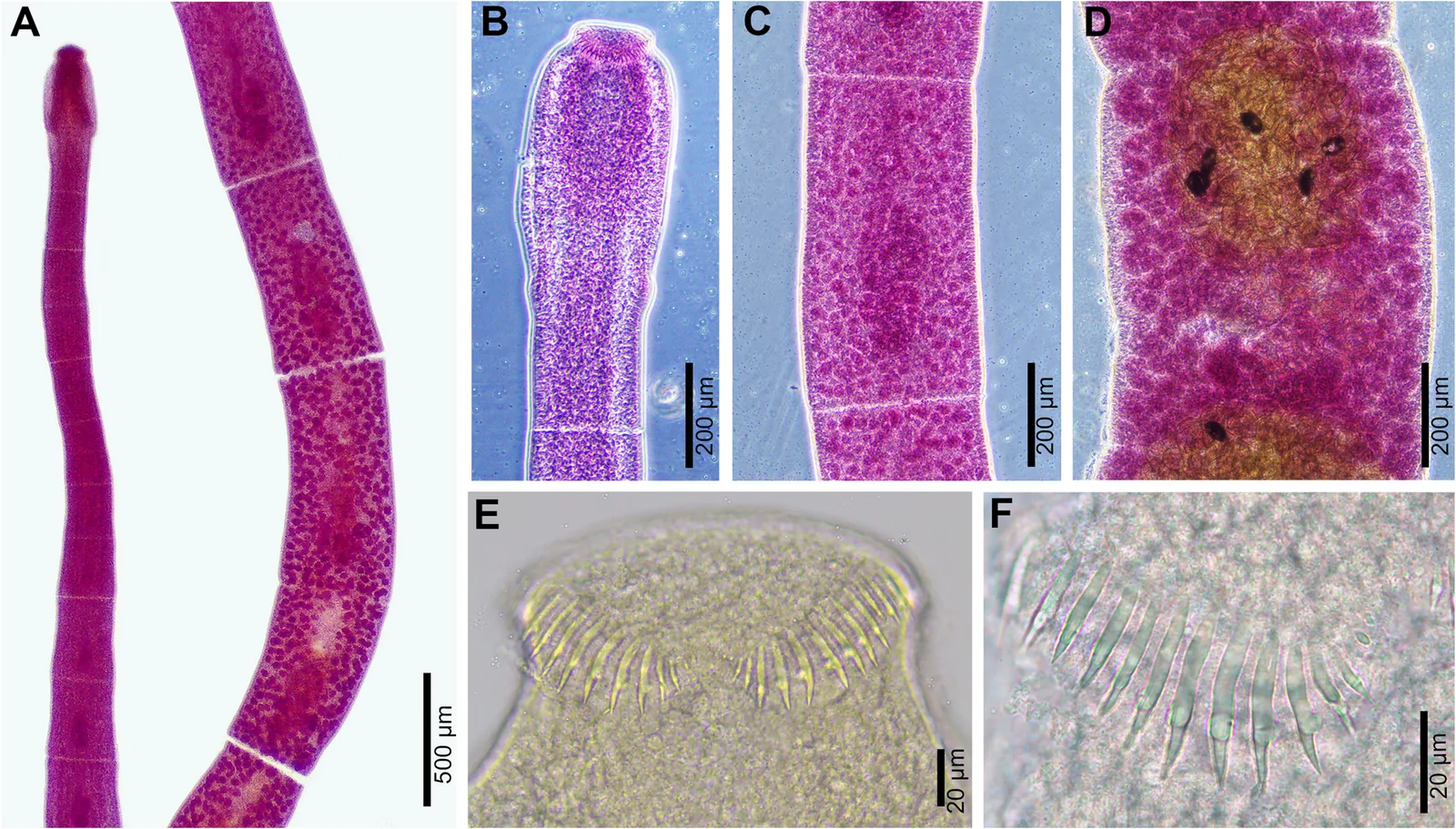
*Senga* sp. found in *Betta splendens* in an ornamental fish farm in Brazil. (**A**) Anterior (left) and mid portion (right) of the parasite. (**B**) Scolex of the type Bothria armed with an apical disc with two half-crowns with hooks. (**C**) Mature proglottid; (**D**) Gravid proglottid. (**E**) Detail of part of the crowns of hooks (ventral view). (**F**) Detail of the hooks (lateral view)

The morphological study of the cestodes obtained in *B. splendens* identified this parasitic agent as *Senga* sp. (Fig. 1). In general, the species reported here is characterized by a rectangular scolex bearing two bothria that occupy 50.3 (44.7–55.0) % of the scolex length. This structure presents an apical disc with two half-crowns bearing 43–44 hooks of different sizes. The larger hook measured 31–35 μm and the small ones 13–16 μm. Moreover, 3–4 rudimentary hooks measuring 5 (4–6) µm can be noted. The mature proglottids are rectangular, acraspedote, with about 100 rounded testes, ovary compact and granular vitellaria scattered throughout all the segment. The uterine pore is median and situated in the first third of the proglottid. The morphology and measures of the scolex and proglottids are compatible with *S. besnardi* (Dollfus 1934) (Table 1). Additionally, the use of the taxonomic key presented by Fernando and Furtado (1963) reveals greater affinity with this species. Despite the morphological similarity to *S. besnardi*, we opt for a genus-level identification due to the lack of molecular evidence supporting conspecificity. Compared with its congeners, our *Senga* sp. and *S. besnardi* can be easily distinguished by the shape of the scolex (rectangular) and relatively small hooks of the apical disc.

**Table 1:** Morphometric data of *Senga* sp. found in *Betta splendens* in an ornamental fish farm from Brazil. Measures from the original description of the species based on worms found in the same fish host are presented for comparison. Data are given by the mean and range between parentheses in micrometers, except for the body length, which is in millimeters

		Present study	Dollfus,1934
		Brazil	France
Boby	L	13.5	15
	W	675 (643–714)	800
Scolex	L	638 (478–821)	687
	W	253 (214–321)	350
Apical disc	L	68 (52–82)	69*
	W	131 (122–148)	153*
Bothria	L	322 (228–428)	375
	W	256 (214–321)	350
Hooks	N	46	46–48
Large hooks	L	33 (32–35)	27.5–35
Lateral hooks	L	21 (19–23)	20.5
Rudimentary hooks	L	5 (4–6)	6–9
Immature proglottids	L	321 (286–357)	362
	W	245 (179–286)	237
Mature proglottids	L	634 (571–678)	NA
	W	386 (336–436)	NA
Gravid proglottids	L	855 (730–1150)	1212
	W	530 (457–584)	575
Eggs	L	44 (44–45)	39–42
	W	27 (24–30)	28–32

*L* length, *W* width, *N* number, *NA* not available. Asterisk indicates measures obtained from the figure

### Discussion and conclusions

This study represents the first record of botriocephalidean cestodes identified as *Senga* sp. in the American continent. Moreover, this agent was associated with a natural occurrence verified in a farm of commercial *B. splendens* in Brazil. The first report of this cestode within a commercial ornamental fish production facility in the country raises significant concerns regarding globalized trade, as this parasite had already been identified in other countries (Dollfus 1934). Consequently, the transport of infected animals between different geographic regions can facilitate the introduction and establishment of novel potentially invasive pathogens in new areas. Therefore, the taxonomic identification and analysis of parasitic indices in one ornamental fish farm will provide unprecedented and fundamental information to prevent the silent dissemination of bothriocephalideans and to protect the economic integrity of Brazilian ornamental aquaculture.

Historically, cestodes previously associated with the genus *Senga* were detected in Brazil in two studies conducted in Campinas, São Paulo state, where parasites were found in the endemic species *Astyanax altiparanae* (Azevedo et al. 2007) and *Astyanax scabripinnis* (Rego 1997). However, specimens obtained by Rego (1997) were restudied by Scholz et al. (2017) and described as a new genus and species, *Regobothrium microhamulinum*. This species clearly differs from *Senga* sp. reported here, especially in scolex morphology. In fact, *R. microhamulinum* is larger (up to 6 cm), presents lower number and different size and shape of the apical disk hooks (14–15 and 7–15 μm). Moreover, the bothria occupy almost the entire length of the scolex (Rego 1997). Regarding the study on *Astyanax altiparanae*, despite reporting the detection of *Senga* sp., the authors did not describe the morphological features of the parasite (Azevedo et al. 2007), precluding any comparison to determine whether it is our *Senga* sp., *R*. *microhamulinum* or a different bothriocephallidean species. These species reported here are morphologically similar to *S. besnardi*, especially in the structure of the scolex (bothria and hooks). However, in the absence of molecular evidence and having more specimens available for new attempts to obtain sequences, we opt not to make a specific identification at this stage of our knowledge. Obtaining sequences of the type species of the genus, *S. besnardi*, will certainly shed more light on current classification of these cestodes. Considering the distinct morphology of *S. besnardi*, it is possible that future phylogenetic studies reveal that other species currently included in *Senga* belong to another genus.

The possibility that the species of *Senga* here reported infects native fish in the natural environment cannot be ruled out. In fact, this phenomenon was verified in *Schyzocotyle acheilognathi (Yamaguti, 1934)*, another cestode species belonging to the family Bothriocephalidae, which was introduced in the Americas in 1975 (Hoffman 1999) and is now widespread across the continent (Choudhury et al. 2006; de Souza et al. 2018). In Brazil, *S. acheilognathi* was identified in a farming system affecting *Cyprinus carpio* (de Souza et al. 2018), as well as in the native fish *Rineloricaria pentamaculata* (de Souza et al. 2018). In Belo Horizonte, Minas Gerais, the same cestode was found in a natural environment parasitizing the ornamental fish *Poecilia reticulata* (Assis and Pinto 2024). In São Francisco do Glória, a municipality located in the same region as the present study, a helminth similar to *S. acheilognathi* was also observed in *Cichlasoma salvini* (Dominguez et al. 2023), which points to the possibility both agents can occur in sympatry. Thus, the differential diagnoses between *Senga* sp. and *S. acheilognathi* should be encouraged after the present study. It is important to note that bothriocephalids exhibit low host specificity to fishes (Scholz et al. 2021), which facilitates the dispersion across different aquatic systems. In this sense, the possibility of the spread of *Senga* sp. in the Americas cannot be ruled out.

Information on the complete life cycle of species of *Senga* is scarce and available only for one species, *Senga visakhapatnamensis* Ramadevi & Hanumantha Rao, 1973, which was studied in India (Ramadevi, 1976). It is here hypothesized that the life cycle of our *Senga* sp. follows the pattern known for this species or even other bothriocephalid cestodes. After non-embryonated eggs are released into the external aquatic environment with the feces of the host, the formation and hatching of the coracidia occur. This moving larval stage is ingested by a copepod that acts as an intermediate host. Within the copepod, the coracidia develop into procercoid. A small fish can act as a second host, in which plerocercoid larvae are formed. Fish that serve as definitive hosts, such as *B. splendens*, probably acquire the parasite after feeding on infected copepods, but the details of this process are poorly known for *Senga* sp. On the evaluated farm, the application of poultry litter as an aquatic fertilizer is a common practice that promotes the accumulation of organic matter. This organic matter serves as a food source for copepods, which subsequently act as vectors for the cestodes present in the rearing environment, thereby facilitating the rapid spread of the parasite throughout the fish culture system. Thus, the implementation of preventive sanitary measures is fundamental to mitigating the spread of diseases in aquaculture. Managing cestodes in their larval stages presents greater challenges than controlling adult forms, necessitating rigorous and periodic parasitological monitoring of ornamental fish. Effective strategies to break the life cycle of the parasite in freshwater environments include sanitary fallowing (periodic drying of the tanks) and disinfection with quicklime (Scholz et al. 2021), aiming to eliminate eggs and intermediate hosts prior to the introduction of a new production batch.

In conclusion, parasitological examination and morphological characterization conducted in this study enabled the identification of *Senga* sp., infecting *B. splendens* in the American continent. This is a potential emerging pathogen of ornamental fish in Brazil, with the possibility of spread across the continent if control measures are not implemented.

## Data Availability

The data will be made available on request.
